# Prevalence and Zoonotic Risk of Multidrug‐Resistant *Escherichia coli* in Bovine Subclinical Mastitis Milk: Insights Into the Virulence and Antimicrobial Resistance

**DOI:** 10.1002/fsn3.4761

**Published:** 2025-01-15

**Authors:** Tonmoy Chowdhury, Mithu Chandra Roy, Ferdaus Mohd Altaf Hossain

**Affiliations:** ^1^ Department of Dairy Science, Faculty of Veterinary, Animal and Biomedical Sciences Sylhet Agricultural University Sylhet Bangladesh; ^2^ Department of Microbial Biotechnology, Faculty of Biotechnology & Genetic Engineering Sylhet Agricultural University Sylhet Bangladesh

**Keywords:** antimicrobial resistance, *Escherichia coli*, Shiga toxin, subclinical mastitis, zoonosis

## Abstract

The emergence of antibiotic‐resistant microorganisms has made antimicrobial resistance a global issue, and milk is a potential source for the propagation of resistant bacteria causing zoonotic diseases. Subclinical mastitis (SCM) cases, often overlooked and mixed with normal milk in dairy farms, frequently involve *E. coli* , which can spread through contaminated milk. We conducted this study to determine the prevalence of virulence genes, antibiotic resistance genes (ARGs), antimicrobial susceptibility, and the genetic relatedness of multidrug‐resistant (MDR) Shiga toxin‐producing *E. coli* (STEC) isolated from SCM milk. SCM‐positive bovine milk was subjected to *E. coli* detection using cultural, biochemical, and molecular methods. Further, we detected STEC virulence genes including stx1, stx2, and eaeA. STEC isolates were tested for ARGs including blaSHV, CITM, tetA, and aac(3)‐IV, and underwent antimicrobial susceptibility tests. Moreover, we performed a phylogenetic analysis of the *stx1* gene of MDR‐STEC. SCM was detected in 47.2% of milk samples of which 50.54% were *E. coli* positive. About 17.20% of *E. coli* isolates contained STEC virulence genes, and *stx2* was the most prevalent. Moreover, all STEC isolates harbored at least one of the ARGs, while about 43.75% of the isolates carried multiple ARGs. Additionally, all the STEC isolates showed multidrug resistance, and were found to be fully resistant against amoxicillin, followed by ampicillin (87.50%) and gentamycin (75%); and were mostly sensitive to aztreonam (81.25%) and meropenem (68.75%). In phylogeny analysis, the *stx1* gene of isolated MDR‐STEC showed close relatedness with disease‐causing non‐O157 and O157 strains of different sources including cattle, humans, and food.

## Graphical abstract

This study highlights the significance of subclinical mastitis milk as a carrier of multidrug‐resistant *Escherichia coli* harboring Shiga toxin virulence genes and antibiotic resistance genes. Furthermore, the Shiga toxin gene in these isolates exhibited a close genetic relationship with disease‐causing strains from diverse sources, emphasizing the potential zoonotic threat.

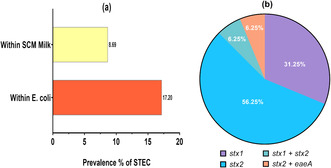

## 1 Introduction

Antimicrobial resistance (AMR) has currently become a matter of worldwide concern in recent decades because of the rapid emergence of antimicrobial‐resistant bacterial strains, particularly *Escherichia coli* ( *E. coli* ), a rod‐shaped gram‐negative bacteria, which is considered a threshold threat to humans, animals, and the environment worldwide, including Bangladesh (Al Amin et al. 2020; Hossain, et al. 2011; Rousham et al. 2018). The Shiga toxin‐producing *E. coli* (STEC) serovars which contain important cytotoxins such as Shiga toxin‐1 (*stx1*), Shiga toxin‐2 (*stx2*), Intimin (*eaeA*), and many more, address intense concerns regarding food safety, antibiotics treatment failure in STEC‐causing diseases, and finally ensuing AMR causing animal and public health hazard (Ansharieta et al. 2021; Farrokh et al. 2013). *E. coli* is considered one of the most important opportunistic bacteria that has been associated with antibiotic resistance and sub‐clinical mastitis (SCM) (Hinthong et al. 2017). Additionally, the potential risk of *E. coli* in SCM, and the presence of harmful STEC have been reported (Ahmadi et al. 2020; El‐Khabaz et al. 2022). Furthermore, excessive antibiotic usage in dairy cows has a substantial impact on the emergence of multidrug resistance in STEC isolates available in milk and dairy products, which soon could result in serious AMR scenarios (Ahmadi et al. 2020; Daini et al. 2005; Mylius et al. 2018).

Cattle itself and raw milk are considered to be reservoirs for STEC bacteria and accountable for *E. coli* O157:H7 infection in humans (Ansharieta et al. 2021; Elafify et al. 2020). Moreover, other disease‐causing non‐O157 serotypes of STEC can also be found in bovine milk (Elhadidy and Mohammed, 2013; Farrokh et al. 2013). The presence of STEC has also been reported in bovine clinical mastitis (visible signs and symptoms) and SCM (without visible signs and symptoms) milk as well (Ahmadi et al. 2020; El‐Khabaz et al. 2022; Momtaz et al. 2012). Usually, hemolytic uremic syndrome (HUS) and hemorrhagic colitis (bloody diarrhea) are the two most common illnesses that occur due to STEC. About 90% percent of STEC‐infected patients develop hemorrhagic colitis, while 5%–15% suffer HUS (Gyles, 2007; Smith et al. 2014; Tarr et al. 2005). STEC‐causing HUS outbreaks in children associated with milk and milk product consumption were reported in France and England (Jones et al. 2019; Treacy et al. 2019). In Bangladesh, enterotoxigenic *E. coli* (ETEC), which is closely linked to STEC (Kaper et al. 2004; Nataro and Kaper, 1998), was estimated to be responsible for 10%–20% of pediatric diarrhea (Qadri et al. 2005). Hence, the presence of STEC in consumable milk and milk products can seriously harm human health.

Moreover, in mastitis, *E. coli* is considered the most common environmental bacterial infection (Eberhart, 1984; Goulart and Mellata, 2022), and also STEC has been reported to be an emerging causal pathogen of bovine mastitis (Murinda et al. 2019). Additionally, the prevalence of multidrug‐resistant (MDR) STEC tends to rise over time (Smith et al. 2014). STEC isolated from bovine mastitis milk already shows resistance against penicillin, streptomycin, tetracycline, ampicillin, gentamycin, etc. antibiotics (Ahmadi et al. 2020; Momtaz et al. 2012). STEC isolated from bovine mastitis milk had been found harboring antibiotic resistance genes (ARGs) such as beta‐lactam class resistance genes (blaSHV, CITM, etc.), tetracycline resistance genes (tetA and tetB), aminoglycoside resistance gene (aac(3)‐IV), and many more (Momtaz et al. 2012). These reports raise concerns about not only the safety of consumable milk and milk products but also bovine udder health which is essential for successful dairying and safe milk production.

Furthermore, SCM in dairy cattle is predominant in Bangladesh and mostly goes unnoticed by the farmer, as a result, the SCM milk gets mixed with normal raw milk in commercial dairy which then goes to market for human consumption (Das et al. 2020; Hasan et al. 2021; Kahir et al. 2008; Rahman et al. 2010). Several factors contribute to SCM in dairy cattle, for example, milking practices addition to other factors like farm hygiene, and feeding may influence the prevalence of SCM (Bari et al. 2022). However, milking practices including machine and hand milking have been reported as an insignificant risk factor for SCM in south asian countries like Srilanka and Bangladesh (Ranasinghe et al. 2021; Tanvi et al. 2024), and in Bangladesh, most farms still practice hand milking (Bari et al. 2022; Das et al. 2016; Shanta et al. 2020). Additionally, milk is supplied to consumers in both raw and processed forms in the dairy value chain in Bangladesh (UNIDO and FAO, 2019). Processed forms include pasteurized milk which is considered to be safer than raw milk, but the pasteurized milk sold in Bangladesh was found to be unsafe with higher bacterial count (Ahmed et al. 2019). This increases the chance of SCM milk containing STEC getting into the human food chain.

In Bangladesh, no study has been conducted on STEC in bovine SCM milk, and minimal data are available on MDR‐STEC and their ARGs concerning bovine SCM milk. So, there is still a huge gap in knowledge on MDR‐STEC in bovine SCM milk. In addition, there is a lack of organized and standard regulations to govern safe milk supply and production. A few initiatives have been taken to improve the condition such as Udder Health Bangladesh (https://uhb.org.bd/) focusing on mastitis research and awareness building, and Food Safety Act Bangladesh (https://bfsa.gov.bd/site/view/law/Food‐Safety‐Act,‐2013) providing quality guidelines for milk and milk products. However, these initiatives are not enough and are yet to be fully implemented across the whole country, specifically in the semi‐urban and rural areas. Hence, we intended to monitor the MDR‐STEC scenario for the bovine SCM milk. These considerations led us to conduct this study to detect the prevalence of STEC in bovine SCM milk. We also identified their virulence and antimicrobial resistance genes, analyzed their antimicrobial resistance profiles, and performed a phylogenetic analysis based on the *stx1* gene to determine their genetic relatedness to other strains.

## 2 Materials and Methods

### 2.1 Sample Collection

**FIGURE 1:**
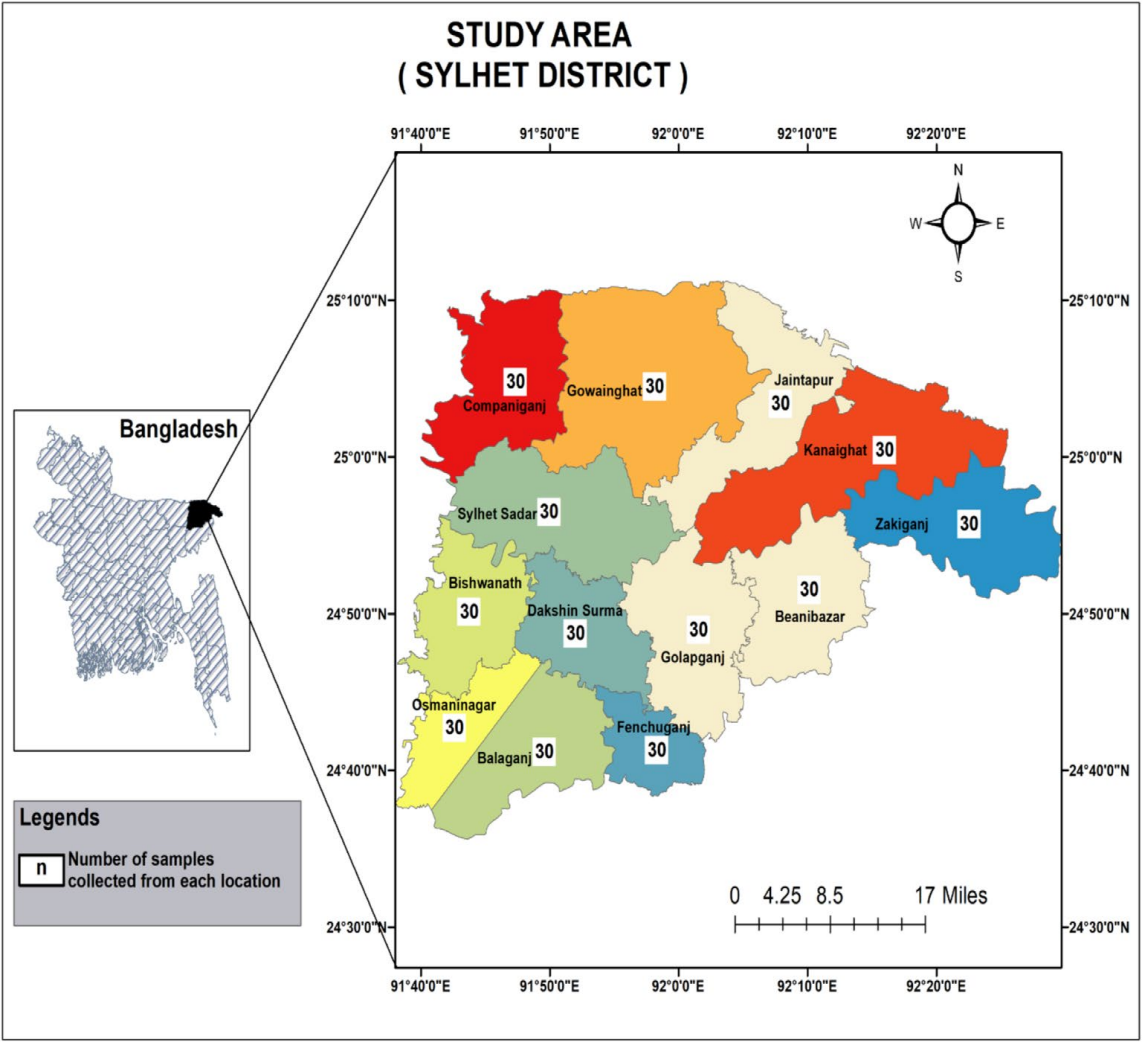
Map of the study area. (A total of 30 milk samples were collected from each upazila. A total number of 390 milk samples were collected from Sylhet district, Bangladesh.)

The samples were collected from 13 upazilas of Sylhet district, Bangladesh (Figure 1). A total of 39 dairy farms from 13 different upazilas (3 farms in each upazila) were randomly selected for the collection of raw milk samples. A total of 390 raw milk samples from individual cows (crossbreed) of randomly selected dairy farms were collected (Figure 1). Milk from all these cows was actively being sold as raw milk by the farms to collectors who supply raw milk to households and the market for consumption and dairy products manufacturing purposes. As hand milking was practiced in all the farms, before collecting the raw milk samples, the milking man's hand and udder were properly cleaned with soap and clean water. The raw milk from all four quarters was then taken directly into a sterilized 15 mL falcon tube. A small amount of raw milk was taken out with a sterilized plastic dropper for conducting the SCM test (Whiteside test). Alongside, the falcon tubes were stored in an airtight icebox and transported as soon as possible to the Dairy Science Laboratory, Department of Dairy Science, Sylhet Agricultural University, Bangladesh for further experiments.

### 2.2 Subclinical Mastitis (SCM) Detection

To detect SCM, the Whiteside test (WST) was performed immediately after the collection of the milk samples at farms. The WST was performed following the descriptions of previous studies (Astermark et al. 1969; Kahir et al. 2008). About 5 drops of milk sample from a falcon tube were taken on a glass slide with a sterilized plastic dropper. Two drops of normal sodium hydroxide (NaOH) solution (4%) were then added to the milk sample on the glass slide. The solution was gently mixed using a glass stirrer. After 20–25 s, if there was the presence of gel formation and/or the solution was broken down into small white flakes, the sample was considered as a SCM positive.

### 2.3 Bacterial Culture

Pre‐enrichment of *E. coli* was done at 37°C for 24 h in Tryptone Soya Broth (TSB) (HiMedia, India). TSB acts as a good medium for enrichment for *E. coli* and has been used in previous studies as well (Reinders et al. 2002; Wallace et al. 1997). For the isolation of the *E. coli* colony, we used Eosin Methylene Blue (EMB) agar (Oxoid Ltd., United Kingdom). EMB agar has been used widely because of its selectivity for gram‐negative bacteria and differential characteristics for *E. coli* where the medium develops colonies with green metallic sheen in the presence of *E. coli* (Antony et al. 2016; Fahim et al. 2019). The inoculated EMB agar was incubated for 24 h at 37°C. Bacterial colonies showing a dark center with a green metallic sheen were presumptively considered *E. coli* colonies. Repeated culture of these presumptive *E. coli* colonies was performed in EMB agar following previously described incubation time and temperature until we got a pure single colony. These single colonies of *E. coli* were further subjected to gram staining and biochemical tests.

### 2.4 Gram Staining of Suspected *E. coli* Colony

A single drop of sterilized distilled water (DW) was taken on the glass microscope slide. Then one single suspected *E. coli* colony was taken with the help of a sterilized platinum loop and mixed with the water drop on the glass microscope slide to dilute the bacterial concentration. After that, the sample was heat‐fixed by passing the glass slide 3–4 times over the flame of the spirit lamp. Next, the gram staining was done following the procedures of TJ Beveridge (2001) (Beveridge, 2001). Finally, the slide was observed at 100X under a light microscope (Model: G‐260, Optima, Taiwan).

### 2.5 Biochemical Tests for *E. coli*

We performed the Motility Indole Urea test (MIU), Methyl Red and Voges‐Proskauer test (MR‐VP), Citrate utilization test (CU), Sugar fermentation test, according to the procedures described by (Feng et al. 2020), and catalase test according to (Karas et al. 2007). The positive result for indole and motility and a negative result for urease was considered as an *E. coli* positive sample (Purkayastha et al. 1970). Development of pink or red color in the medium on the Methyl‐Red (MR) test was considered as *E. coli* positive characteristics (Karim et al. 2020) while *E. coli* giving a negative reaction (no color development) for the VP test was considered as *E. coli* positive (Karim et al. 2020). As the development of blue color in the CU test indicates a positive growth reaction, so *E. coli* showing a negative reaction (no color development) in CU was considered as an *E. coli* positive sample (Dash et al. 2012). Moreover, the production of gas and media color changing into yellow in the Sugar fermentation test were considered characteristics of the *E. coli* positive sample (Dash et al. 2012). Furthermore, in the case of the catalase test, if there was a presence of bubbling within the 1‐min observation time then the sample was considered catalase positive, and if there was no bubbling throughout the one‐minute observation, the sample was interpreted as catalase negative.

### 2.6 Preservation of *E. coli* Isolates

The EMB culture samples that showed characteristics of *E. coli* and confirmed in gram staining and biochemical tests were preserved for further use. Preservation was done using nutrient broth (NB) and glycerol (15%) mixed solution described by a previous study (Gorman and Adley, 2004). To stock the bacterial cultures, firstly NB (HiMedia, India) was prepared by dissolving at an amount of 13 g in 1000 mL DW and autoclaved at 121°C for 15 min at 15 psi of pressure for sterilization. After completing the autoclave process, the NB was kept in a water bath to cool down at a temperature of 37°C and distributed at an amount of 10 mL in each test tube. Then a single colony of *E. coli* from EMB agar was streaked into sterilized NB containing test tubes. The test tubes were closed using cotton plugs and then incubated at 37°C for 24 h. After incubation, the growth of bacteria was confirmed by the development of turbidity in the broth. Finally, for preservation, 850 μL of broth culture with 150 μL of sterile 100% glycerol was kept in a sterilized 1.5 mL Eppendorf tube and gently inverted two times to mix. The Eppendorf tubes were marked with respective sample IDs and kept at −20°C for future use.

### 2.7 Bacterial DNA Extraction

Extraction of bacterial DNA was done by the boiling method as described in a previous study (Md. S. Islam et al. 2021). However, we slightly modified some steps in the method but didn't alter the basic steps of boiling method DNA extraction (Md. S. Islam et al. 2021). Firstly, Nutrient agar (NA) (HiMedia, India) was prepared by adding and dissolving NA at an amount of 28 g in 1000 mL DW in a sterilized conical flask. The medium was then autoclaved at 121°C for 15 min at 15 psi of pressure. After autoclaving, the conical flask was kept in a water bath to cool down to 45°C and then spread on a petri dish inside a laminar airflow cabinet (Biobase, China). When the NA medium solidified in the petri dish, then the preserved cultures of *E. coli* isolates were streaked on the NA medium using a sterilized platinum loop. The petri‐dish were then incubated at 37°C for 24 h. After the incubation period, the medium‐sized colonies were taken into nuclease‐free water (500 μL) in an Eppendorf tube (Hyclone Laboratories Inc., USA) and the Eppendorf tube was vortexed for 10 s using a vortex machine (Model: MS‐S, DLAB, China). After that, to pellet the contents of Eppendorf tubes, centrifugation was done at 13000 rpm for 5 min using a centrifuged machine (Model: D3024, DLAB, China). The supernatants were discarded from the Eppendorf tubes. The tubes were again filled with 500 μL nuclease free water and vortexed for 5 s. Following that, the tubes were then placed on boiling water in the water bath for 10 min. After that, all the Eppendorf tubes were immediately kept on −20°C for 10 min. After cooling, the Eppendorf tubes were subjected to centrifugation at 13000 rpm for 5 min. Finally, 50 μL of supernatant from Eppendorf tube was moved to another sterilized Eppendorf tube and used as DNA template. The DNA templates were stored at −20°C for further use.

### 2.8 Molecular Detection of *E. coli*, STEC, and ARGs by PCR

PCR was conducted for molecular detection of *E. coli* (16 s rRNA), STEC genes (stx1, stx2, eaeA), and ARGs (blaSHV, CITM, tetA, aac(3)‐IV) respectively. PCR was performed on a thermal cycler (Model: T100, Bio‐Rad Laboratories Inc.). For the PCR reaction mixture, we used Emerald Amp MAX PCR Master Mix (2X) (Takara Bio, USA). All the primer sets were manufactured by Macrogen Inc. (Seoul, South Korea). The primers used in our experiments were taken from previous studies (Dipineto et al. 2006; Guillaume et al. 2000; Hessain et al. 2015; Khan et al. 2002; Parvin et al. 2020; Van et al. 2008), and the details are mentioned in (Table 1). PCR tubes (0.2 mL volume) were used for performing PCR. For preparing PCR reaction mixtures, at first PCR tubes were autoclaved and then kept inside a laminar airflow cabinet maintaining aseptic condition. The PCR reaction mixture was then prepared at a volume of 25 μL. We added 12.5 μL of master mix, 1 μL (10 picomoles per 1 μL concentration) of forward primer, 1 μL (10 picomoles per 1 μL concentration) of reverse primer, 4 μL of DNA template, and 6.5 μL of nuclease‐free water (Hyclone Laboratories Inc., USA). The PCR conditions were set to initial denaturation at 95°C for 5 min and final extension at 72°C for 7 min for all the primers. The number of cycles, temperature, and time for denaturation, annealing, and extension steps were different for each primer and have been stated in (Table 1).

**TABLE 1:** Primers used in molecular detection of *E. coli* , STEC, and ARGs.

	Target gene	Nucleotide Sequences (5″–3″)	PCR condition				Amplicon size (bp)	Reference
			Denaturation	Annealing	Extension	Cycles		
*Escherichia coli*	malB promoter (16 s‐rRNA)	F‐ GACCTCGGTTTAGTTCACAGA R‐ CACACGCTGACGCTGACCA	94°C–1 min	58°C–1 min	72°C–45 s	35	585	Parvin et al. 2020
Shiga toxin‐producing *Escherichia coli* (STEC)	stx1	F‐ CAACACTGGATGATCTCAG R‐ CCCCCTCAACTGCTAATA	94°C—1 min	55°C–1 min	72°C–30s	35	349	Hessain et al. 2015
	stx2	F‐ CCATGACAACGGACAGCAGTT R‐CCTGTCAACTGAGCAGCACTTTG	94°C—1 min	58°C–40s	72°C–1 min	35	779	Dipineto et al. 2006
	eaeA	F‐ AAACAGGTGAAACTGTTGCC R‐ CTCTGCAGATTAACCTCTGC	94°C—1 min	58°C–1min	72°C–40s	35	350	Khan et al. 2002
Antibiotic resistance genes (ARGs)	blaSHV	F‐ TCGCCTGTGTATTATCTCCC R‐ CGCAGATAAATCACCACAATG	94°C—1 min	55°C– 1 min	72°C–1 min	35	768	Van et al. 2008
	CITM	F‐ TGGCCAGAACTGACAGGCAAA R‐ TTTCTCCTGAACGTGGCTGGC	94°C—1 min	55°C– 1 min	72°C–40s	35	462	Van et al. 2008
	Tet(A)	F‐ GGCCTCAATTTCCTGACG R‐ AAGCAGGATGTAGCCTGTGC	94°C—1 min	55°C– 1 min	72°C–30s	35	372	Guillaume et al. 2000
	aac(3)‐IV	F‐ CTTCAGGATGGCAAGTTGGT R‐ TCATCTCGTTCTCCGCTCAT	94°C—1 min	55°C– 1 min	72°C–30s	35	286	Van et al. 2008

Abbreviations: bp, base pair; F, Forward; min, minute; R, Reverse; s, seconds.

### 2.9 Gel Electrophoresis

To perform gel electrophoresis, 50 mL of 1.5% agarose solution was prepared by mixing and dissolving 0.75 g ultrapure agarose powder (Invitrogen, Life Technologies, USA) and 1X TAE buffer (Himedia, India). The solution was then cooled down at 60°C–70°C and 0.5 μL of Ethidium bromide 1% solution (Carl Roth GmbH + Co. KG, Germany) was added to the agarose solution. The solution was then gently mixed and poured on the side of the gel plate where the comb was placed and left for 15 min to solidify. After solidification of the gel, the gel was carefully placed on the electrophoresis tank (BTLab systems, A Geno Technologies Inc., USA), and submerged about 5–6 mm in 1X TAE buffer. The PCR products (5 μL) were then loaded in the well of the gel. 5 μL of 100 bp DNA ladder showing each band increment from 100 bp to 1000 bp and a 1500 bp band (Eco in action, GeneDirex Inc., South Korea) was also loaded on a gel well. The gel electrophoresis was then performed at 100 V for 30 min using a gel electrophoresis system (BTLab Systems, A Geno Technologies Inc., USA). After completion of electrophoresis, the gel was then taken out of the gel tank and visualized under the gel documentation system using UV trans‐illumination (Bio‐Rad Laboratories Inc., USA).

### 2.10 Antimicrobial Susceptibility Test

The antimicrobial susceptibility test of *E. coli* was performed following the disc diffusion method as described in previous studies and guidelines (Bauer et al. 1966; CLSI, 2020). Mueller‐Hinton agar (Himedia, India) was prepared by dissolving the agar medium at an amount of 38 g in 1000 mL of DW and sterilized by autoclaving at 121°C for 15 min at 15 psi of pressure. After autoclaving, the media was then kept in a water bath to cool down to 39°C. Then the media was poured onto a petri dish and left in the laminar airflow cabinet until the media solidified. Then colonies of *E. coli* isolates which were cultured and incubated (24 h at 37°C) in nutrient agar (HiMedia, India), were suspended in 3 mL of normal saline (0.85% NaCl solution) and matched to 0.5 McFarland standards. The samples were then dispersed using an L‐shaped spreader onto the Mueller‐Hinton agar medium. After that antibiotic discs (HiMedia, India) were placed on the agar medium. The Petri dishes were then kept on incubation for 24 h at 37°C. After the completion of the incubation period, the inhibition zones were measured according to the standard guidelines of a previous study (CLSI, 2020). Bacteria were considered MDR when the bacteria showed resistance against at least 1 antimicrobial of 3 or more different classes of antimicrobials (Magiorakos et al. 2012; Rozwadowski and Gawel, 2022; Sweeney et al. 2018). We used 13 antibiotics for the antimicrobial susceptibility test (Table S1).

### 2.11 PCR Amplicon Sequencing and Phylogenetic Analysis

The amplified PCR product (25 μL) of the *stx1* gene of an MDR‐STEC isolate was subjected to Sanger DNA sequencing (Sanger et al. 1977) at the Genome Center, Jashore University of Science and Technology, Jessore, Khulna, Bangladesh. To perform phylogenetic analysis, a total of 57 nucleotide sequences of the *stx1* gene of *E. coli* isolates were retrieved from the NCBI (National Center for Biotechnology Information) database. Isolation sources of these isolates were cattle, beef, cheese, cattle feces, human, and human stool. Multiple sequence alignment was done using MEGA 11 (Molecular Evolutionary Genetics Analysis) software. The best model fit was K2P (unequal transition/transversion rates and equal base frequency), which was determined by using ModelFinder in IQ‐Tree (Kalyaanamoorthy et al. 2017). The phylogenetic tree was constructed based on the maximum likelihood method on IQ‐Tree version 2.3.4 (Nguyen et al. 2015). Furthermore, Fig Tree software and Microsoft PowerPoint 2019 were used for annotating the phylogenetic tree.

### 2.12 Statistical Analysis and Data Visualization

All quantitative data was recorded on Microsoft Excel 2019. The GraphPad Prism 9.3.1 statistical software was used for descriptive analysis and generating graphs.

## 3 Results

### 3.1 Prevalence of Bovine SCM

**FIGURE 2:**
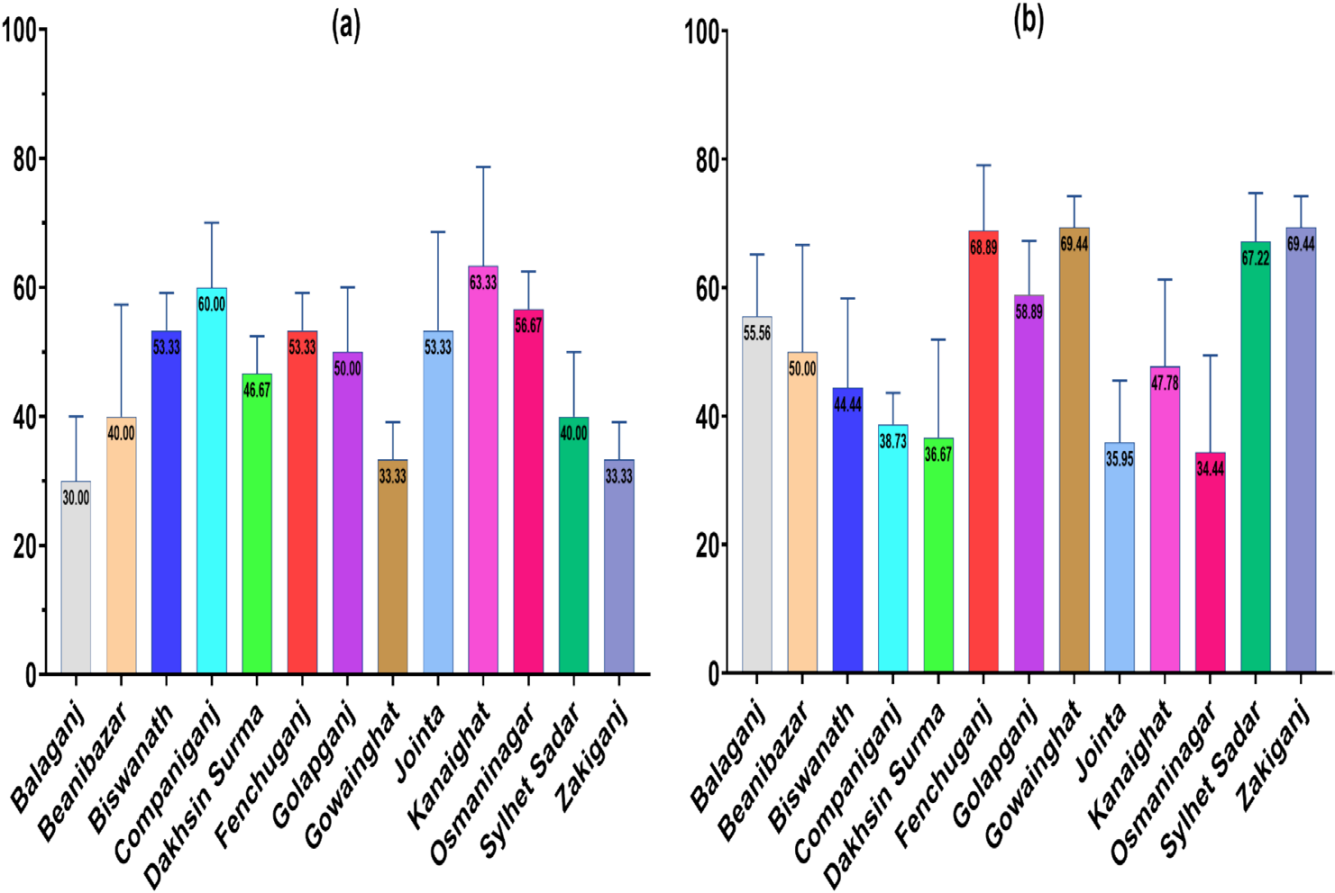
Prevalence of SCM in milk samples and detection of *E. coli* in SCM‐positive samples. (The bar graph (a) shows the SCM prevalence in dairy cattle in different upazilas of Sylhet. The total number of SCM‐positive samples was 184 out of 390 milk samples. The graph (b) depicts the prevalence of *E. coli* in SCM‐positive samples. About 93 out of 184 SCM‐positive samples were identified as *E. coli* ‐positive samples.)

The overall prevalence of bovine SCM was found about 47.2% (184 out of 390 milk samples) in Sylhet district with a lowest mean percentage of 30% in Balaganj upazila and a highest of 63.3% in Kanaighat upazila (Figure 2a).

### 3.2 Prevalence of *E. coli*

All culture and biochemical characteristics that were found in this study for *E. coli* ‐positive samples are given in the supplementary file (Table S2). The percentage of culture‐positive presumed *E. coli* was found in 110 samples out of 184 SCM‐positive samples. About 101 biochemical test‐positive *E. coli* samples were found in the culture‐positive samples. Among those, a total of 93 samples were found *E. coli* positive in molecular detection. Hence the total percentage of *E. coli* positive samples was 50.54% (93 out of 184) in SCM positive samples. The lowest mean percentage of *E. coli* presence in SCM milk samples was 34.44% in Osmaninagar upazila, while the Gowainghat and Zakiganj upazilas had the highest percentage of 69.44% (Figure 2b).

### 3.3 Prevalence of STEC and Virulence Genes

**FIGURE 3:**
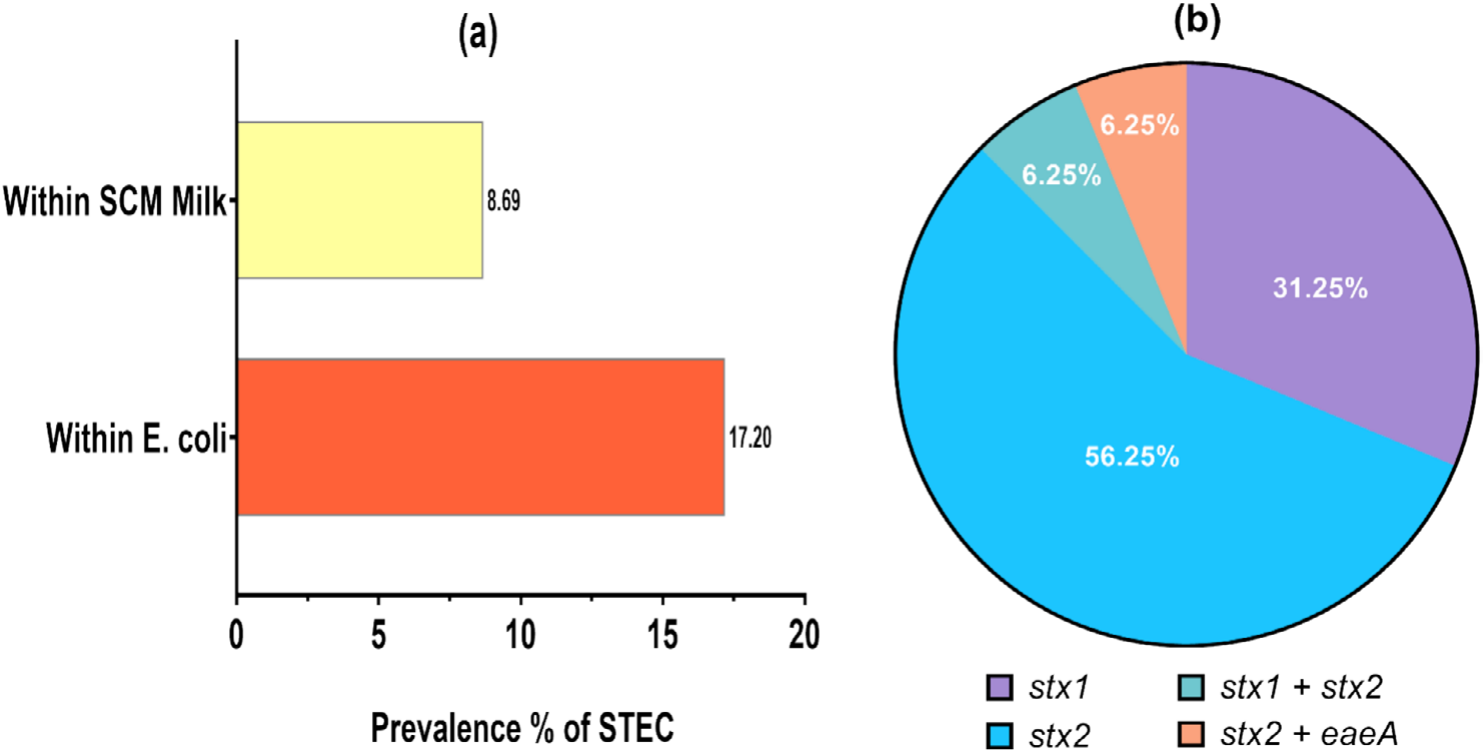
Prevalence of STEC and detection of virulence genes in STEC. (Graph (a) illustrates the prevalence of STEC in 184 SCM‐positive milk samples and 93 *E. coli* ‐positive SCM milk samples. The pie chart (b) shows the percentages of virulence genes found in STEC samples (*n* = 16).)

We found a total of 16 samples as STEC‐positive. Hence, the prevalence of STEC in *E. coli* ‐positive samples was 17.20% (16 out of 93), and the prevalence of STEC in the SCM milk samples was 8.69% (16 out of 184 SCM samples) (Figure 3a). About 31.25% (*n* = 5) STEC was found to contain *stx1* gene, and 56.25% (*n* = 9) STEC had *stx2* gene. But no sample was found positive for only the *eaeA* gene. We found 6.25% (*n* = 1) STEC to be harboring both *stx1* and *stx2* genes, and 6.25% (*n* = 1) STEC harboring both *stx2* and *eaeA* genes (Figure 3b).

### 3.4 Molecular Detection of ARGs in STEC

**FIGURE 4:**
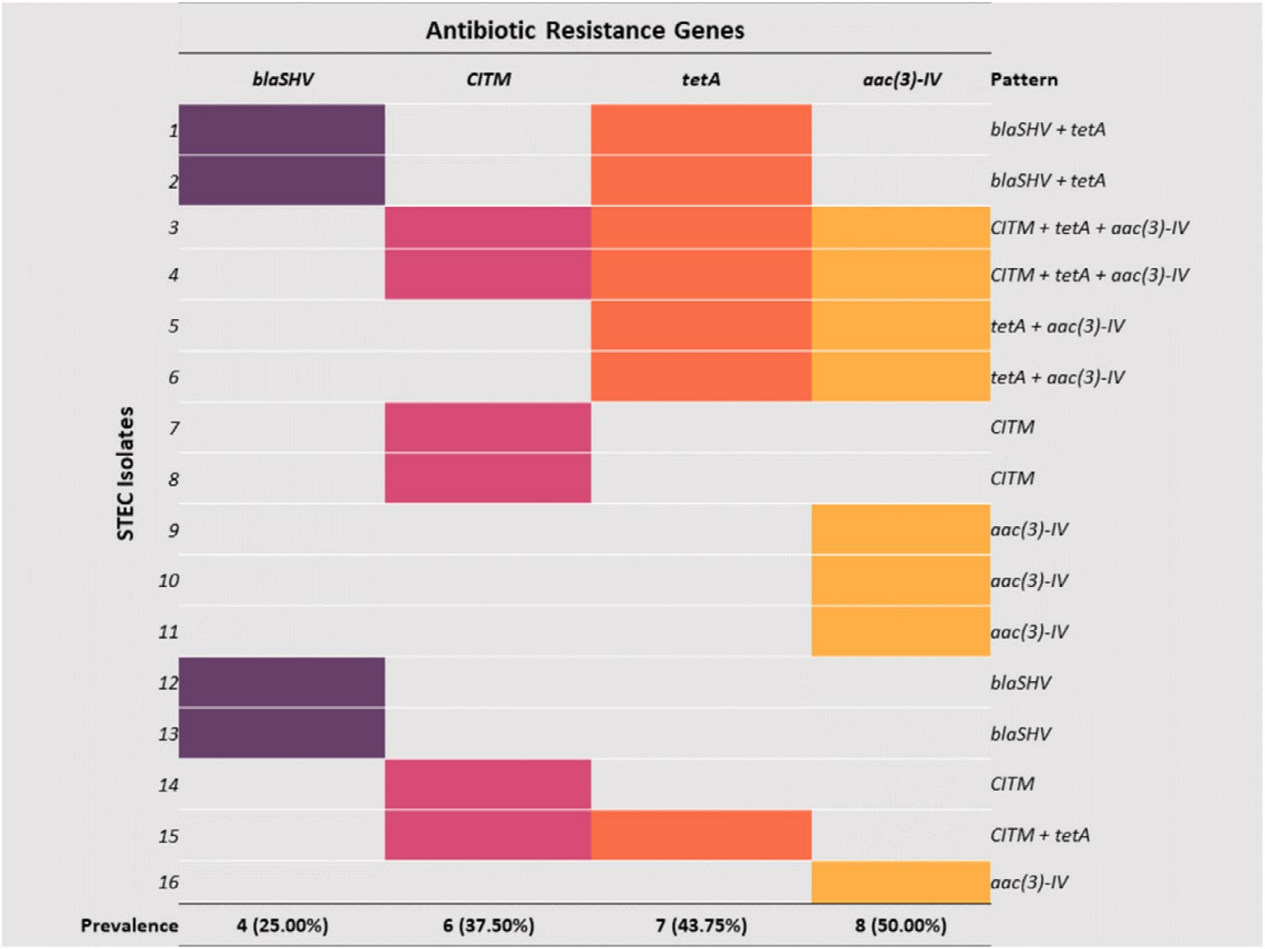
Prevalence of AGRs in STEC samples. (The left side shows 16 different STEC isolates and the right side shows the pattern of AGRs present in each isolate.)

In case of ARGs, all STEC isolates (*n* = 16 out of 16) carried at least one of the ARGs, and among them 25.00% (*n* = 4) harbored blaSHV gene, 37.50% (*n* = 6) contained CITM gene, 43.75% (*n* = 7) had tetA gene, and 50.00% (*n* = 8) carried aac(3)‐IV gene (Figure 4). The ARGs patterns among the STEC were found to be blaSHV + tetA (*n* = 2), CITM + tetA+ aac(3)‐IV (*n* = 2), tetA + aac(3)‐IV (*n* = 2), and CITM + tetA (*n* = 1); that revealed about 43.75% (*n* = 7/16) STEC isolates carried multiple antibiotic resistant genes (Figure 4).

### 3.5 Antimicrobial Susceptibility of STEC

**FIGURE 5:**
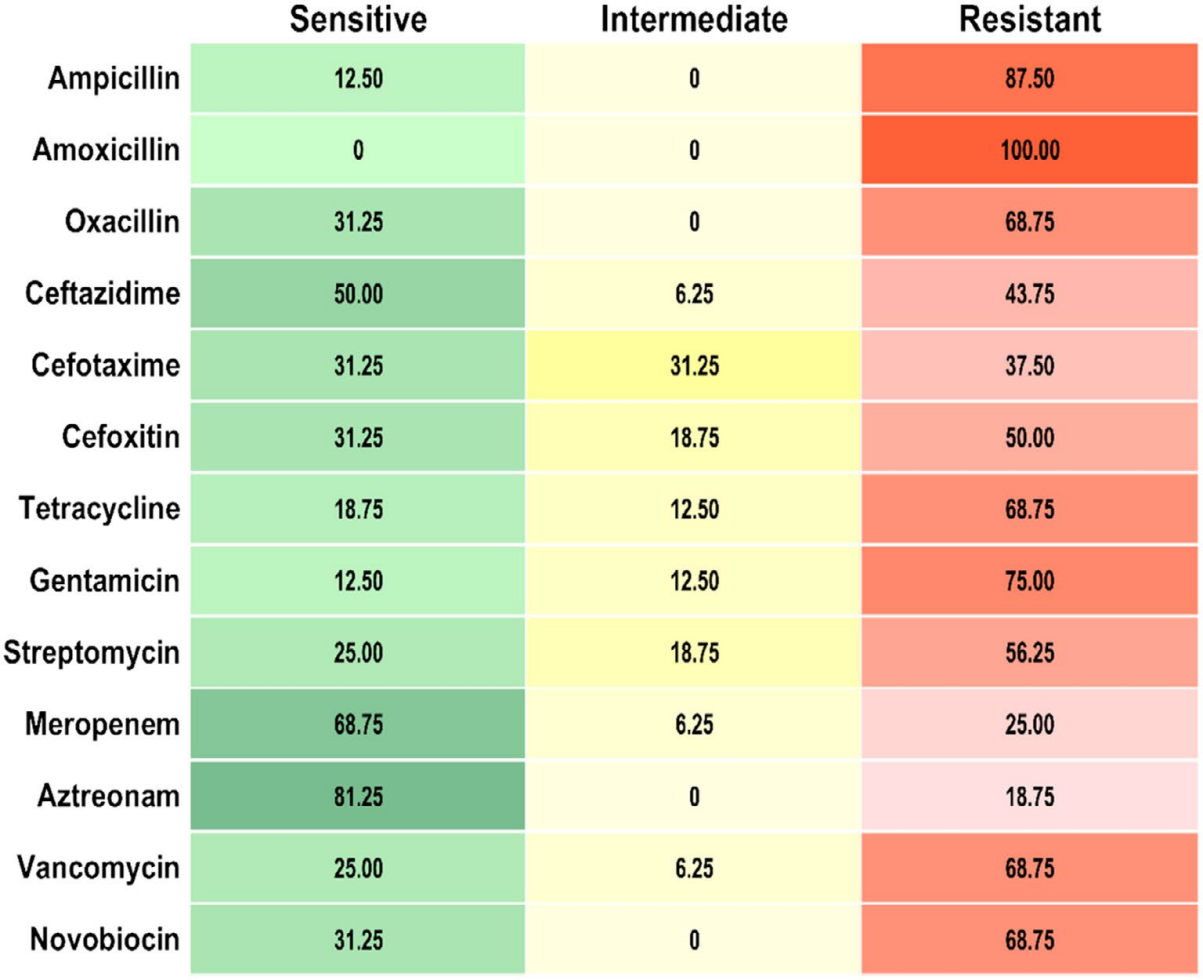
Antibiotic resistance profile of STEC isolates (*n* = 16) isolated from SCM milk.

Antimicrobial susceptibility test by disc diffusion method revealed that all STEC isolates (*n* = 16) were MDR (Figure 5). All of them were resistant to amoxicillin (100%). The second highest resistance was recorded against ampicillin (87.50%), followed by gentamycin (75%), tetracycline (68.75%), vancomycin (68.75%), and novobiocin (68.75%) (Figure 5). On the other hand, STEC isolates were mostly sensitive to aztreonam (81.25%) and meropenem (68.75%) (Figure 5).

### 3.6 Phylogenetic Analysis of MDR‐STEC Isolate

**FIGURE 6:**
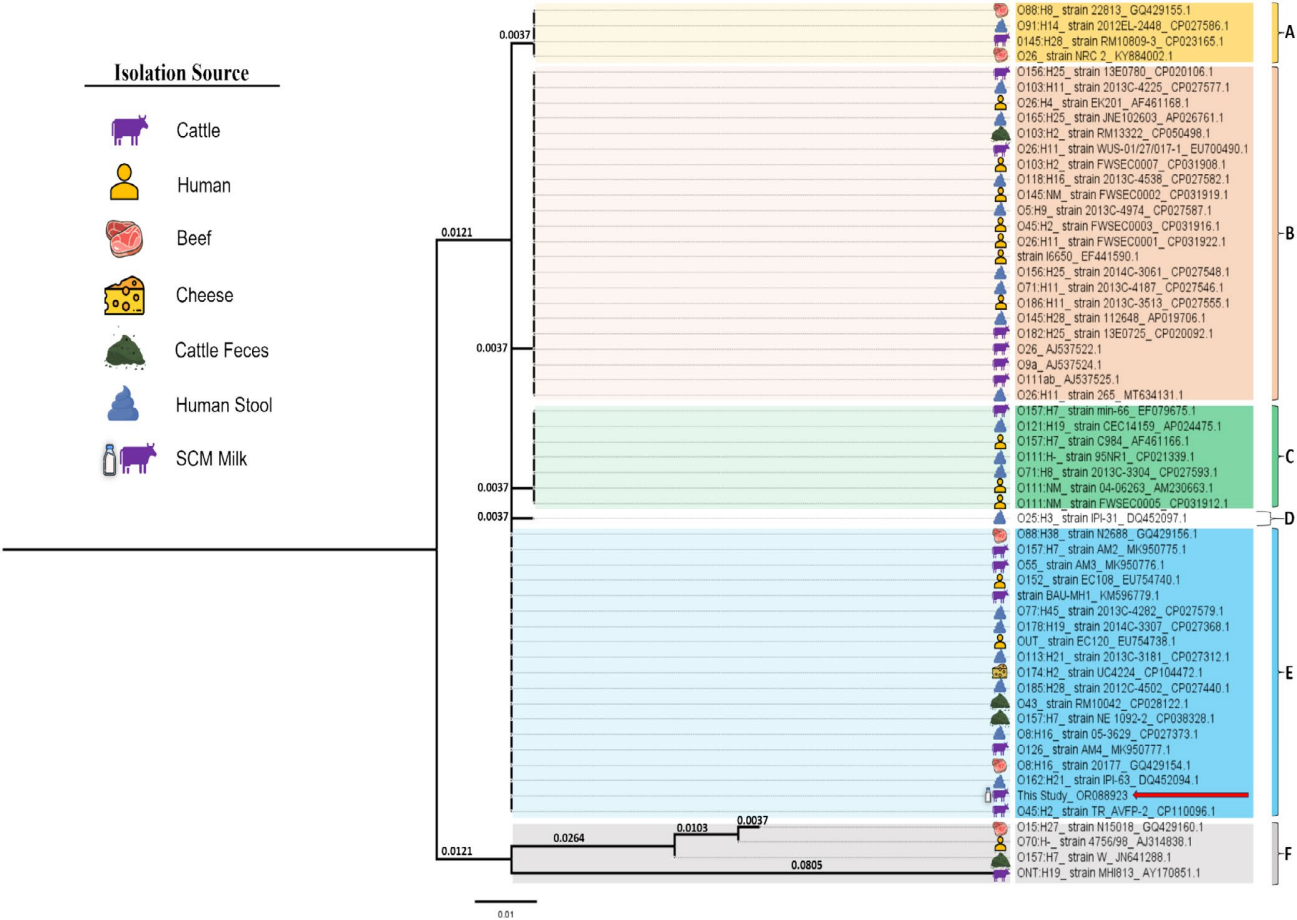
Phylogenetic analysis of stx1 gene of MDR‐STEC isolated from SCM milk. (The red arrow shows the stx1 gene sequence (NCBI accession no.: OR088923) of the current study. Six different groups (A to F) based on genetic distance are depicted in different colors. The isolation sources of different sequences are illustrated adjacent to the sequence labels.)

Phylogenetic analysis of amplified DNA sequence of the *stx1* gene of MDR‐STEC revealed that our study sequence (NCBI accession no.: OR088923) was closely related to the *stx1 gene* of various *E. coli* serotypes in Group E including both O157:H7 and non‐O157:H7 isolated from various sources (Figure 6). However, most of the strains with close relativeness with our study sequence were non‐O157:H7 and only two were O157:H7. Among a total of 18 of these *E. coli* serotypes, mostly were from human stool source (*n* = 6) and cattle source (*n* = 5), followed by beef (*n* = 2), cattle feces (*n* = 2), cheese (*n* = 1), and human clinical sample (*n* = 2) (Figure 6). Other groups such as groups A, B, C, and D were distantly related to our study sequence compared to group E, whereas group F was the most distant among all the groups (Figure 6).

## 4 Discussion

Mastitis has a serious financial impact on dairy farming worldwide. SCM is particularly highlighted because there are no obvious visible signs to diagnose (Momtaz et al. 2012). In the current study, we found about 47.2% of the milk samples of crossbreed dairy cattle to be SCM positive with the lowest range of 30% and the highest range of 63.3% (Figure 2a). A similar trend in the prevalence of bovine SCM in Sylhet had been reported in previous studies, where prevalence was found to be 42%–54% (Das et al. 2020; Hasan et al. 2017; Kahir et al. 2008; Rahman et al. 2010). Along with that, SCM is not only highly prevalent in current study areas but also prevalent (51%–67.9% SCM prevalence) across the country (Hasan et al. 2021; Kabir et al. 2017; Kayesh et al. 2014; Rabbani and Samad, 2010). However, in very few regions of Bangladesh, the prevalence of SCM is found to be below 30% (Islam et al. 2011; Sarker et al. 2013). These previous reports and current study findings indicate that the SCM prevalence remained constant over time. This scenario can be associated with unhygienic practices and unawareness of the risk factors among dairy farmers. One study conducted in Bangladesh that directly observed the milking practices in farms reported around 60% of the dairy farmers did not wash their hands before milking and the rest used only water to wash their hands (Shanta et al. 2020). Additionally, only about 33% of the farmers washed cow udder with normal water and 100% of them did not practice teat dipping and wiping udder after milking (Shanta et al. 2020). Another study found that while farmers self‐reported washing hands and udder and were aware of SCM, fewer practiced proper floor cleaning, none performed SCM tests in their farms, and all farms showed significantly higher SCM prevalence (Dey et al. 2023). Moreover, a study on dairy farms in Bangladesh found that around 82% of the farms had poor levels of biosecurity, health, and disease management practices (Bushra et al. 2024). This situation indicates that the dairy farmers are negligent regarding the risk of SCM, and the measures needed for minimizing the risk factors of SCM are not being properly implemented.

Moreover, in SCM conditions, the udder appears normal without any detectable clinical signs (Girma and Tamir, 2022). As a result, farmers are unable to isolate the SCM‐infected cows and usually, the SCM milk is mixed with normal milk when collected in bulk tanks to supply for human consumption or different milk products manufacturing. Additionally, considering *E. coli* is a predominant environmental bacteria in bovine mastitis condition (Eberhart, 1984; Goulart and Mellata, 2022), and STEC being an emerging bacteria for SCM (Murinda et al. 2019) increases the concern of STEC contamination in consumable milk and dairy products which may pose public health risks. In our study, the number of *E. coli* ‐positive samples found in cultural and biochemical tests were 110 and 101 out of 184 SCM milk samples, respectively. The cultural and biochemical tests may give false positive results as some non‐ *E. coli* bacteria can imitate *E. coli* characteristics (Antony et al. 2016). However, molecular detection revealed *E. coli* isolates prevalence of about 50.54% (*n* = 93 out of 184) in SCM milk (Figure 2b). Previous studies reported that *E. coli* prevalence in SCM milk ranged from 16% to 58.78% (Ahmad et al. 2021; Farhad et al. 2021; Faruk Siddiki, 2019; Haque et al. 2014; Momtaz et al. 2012). Therefore, normal milk mixed with SCM milk which has a predominant presence of *E. coli* is a serious food safety breach that exists in the current dairy food chain in Bangladesh. Furthermore, the concern of contamination gets more complex if there is a presence of STEC.

The STEC is considered to be responsible for causing life‐threatening diseases like hemorrhagic colitis and HUS (Smith et al. 2014). STEC is also considered a significant contributor to kidney failure in children (Farrokh et al. 2013). Hence, consumable milk, and milk products that are contaminated with STEC are a serious concern. In our study, 17.20% (*n* = 16 out of 93) isolates of *E. coli* had at least one STEC virulence gene (*stx1*, *stx2*, and *eaeA*), and the STEC prevalence in SCM milk was 8.69% (*n* = 16 out of 184) (Figure 3a). Moreover, in Bangladesh, the milk producers maintain a poor level of hygiene (Rana et al. 2023; Shanta et al. 2020) and the pasteurized milk from different brands was found to be contaminated with a high bacterial count which indicates a poor food safety practice at processing plants (Ahmed et al. 2019). Hence, the prevalence of STEC in SCM milk indicates that there is a subtle concern about suffering STEC infection if someone consumes milk or milk products that have been made out of milk that had SCM milk mixed in it. In Bangladesh, 2.05% and 2.15% of STEC prevalence in raw milk were identified (Alam et al. 2021; Islam et al. 2010). So, SCM milk is more likely than raw milk to be contaminated with STEC. Unfortunately, no reliable study on STEC in SCM milk was found in Bangladesh, but similar studies in other countries reported STEC prevalence in SCM milk as 9% in Egypt (El‐Khabaz et al. 2022), 9.7% and 27.23% in Iran (Ahmadi et al. 2020; Momtaz et al. 2012), 9.5% in Brazil (Rangel and Marin, 2009), and 32% in Nigeria (Ivbade et al. 2014). Though our study shows slightly less prevalence of STEC compared to other countries, still this needs to be considered as an emerging threat to public health risks.

Moreover, we detected 31.25% of STEC isolates carrying the stx1 gene, 56.25% of isolates containing the stx2 gene, and 6.25% of isolates carrying both stx1 and stx2 (Figure 3b). Additionally, no eaeA (intimin) gene was found to be present individually, but 6.25% of isolates carried stx2 and eaeA combinedly (Figure 3b). The stx1 and stx2 genes are the most important virulence factors of STEC, and the eaeA gene is considered a complementary virulence factor of STEC in human illness but its mode of action in dairy cattle is yet to be defined (Ahmadi et al. 2020; Mahanti et al. 2013). Among these virulence genes, the stx2 is more virulent than stx1, and the stx2 is mostly related to hemorrhagic colitis, and HUS (Ahmadi et al. 2020). Our overall study result shows that stx2 has a higher prevalence in SCM milk (Figure 3b). However, the prevalence of stx2 and stx1 genes has been observed to vary. For instance, a previous study on STEC in SCM milk in Iran reported a higher prevalence of stx2 (Ahmadi et al. 2020), while another study in the same country found a higher prevalence of stx1 in STEC isolated from SCM milk (Momtaz et al. 2012). However, in Bangladesh, the stx2 gene was reported to be predominant in STEC isolated from normal raw milk (Alam et al. 2021). The presence of STEC harboring stx1, and stx2 can cause serious health problems if transmitted to humans. In Bangladesh, enterotoxigenic *E. coli* (ETEC), which is closely linked to STEC (Kaper et al. 2004; Nataro and Kaper, 1998), was estimated to be responsible for 10%–20% of pediatric diarrhea (Qadri et al. 2005). Additionally, outbreaks of STEC that caused HUS in children linked to milk, and milk product consumption have been reported in France, and England (Jones et al. 2019; Treacy et al. 2019). Along with that, if the STEC exhibits antibiotic resistance, then the health and food safety problem may reach a new level of perplexity.

Furthermore, the AMR emergence in animal‐origin food and food products due to the use of indiscriminate antibiotics usage is a significant issue for the safety of both human and animal health (Al Amin et al. 2020; Rousham et al. 2018). In the current study, all of the STEC isolates had at least one of the four (blaSHV, CITM, tet(A), and aac(3)‐IV) ARGs (Figure 4). The most prevalent antibiotic‐resistant gene was gentamycin‐resistant gene aac(3)‐IV (50%), followed by tetracycline‐resistant tetA gene (43.75%), ESBL‐resistant CITM (37.50%) and blaSHV (25%) (Figure 4). Unfortunately, no reliable data could be found on the prevalence of ARGs in STEC isolated from bovine SCM milk in Bangladesh. One study conducted in Bangladesh regarding clinical mastitis milk found the tet(A) gene to be present in all the non‐STEC *E. coli* isolates but no presence of blaSHV (Bag et al. 2021); these findings cannot be compared to our findings as isolates of the previous study were STEC negative (Bag et al. 2021). However, one previous study reported a prevalence of ARGs in STEC isolated from bovine mastitis milk in Iran—aac(3)‐IV (27.65%), CITM (12.76%), and blaSHV (6.38%)—which were lower than our findings, but the prevalence of tet(A) (48.93%) was higher compared to our study (Momtaz et al. 2012). The ARGs prevalence in STEC may differ due to several factors, such as the predominant use of particular antibiotics in the specific study region; other than that, forage, farm location, management practice, and even frequent contact with other animals can also influence the prevalence of ARGs, as these factors can play a crucial role in adaption and transmission of ARGs (Liu et al. 2024). The spread of antibiotic‐resistance genes from *E. coli* pathotypes like STEC to other pathogenic or commensal strains is another aspect of the risk in the public and dairy sectors (Ahmadi et al. 2020; Dormanesh et al. 2014). Moreover, we found STEC isolates were harboring multiple ARGs genes in different combinations such as blaSHV + tetA, CITM + tetA+ aac(3)‐IV, tetA + aac(3)‐IV, and *CITM +* tetA (Figure 4). The presence of multiple ARGs in STEC was reported by a previous study as well (Momtaz et al. 2012); the combination pattern or prevalence of different ARGs may change depending on serotypes (Momtaz et al. 2012). In our study, the pattern of ARGs could not be strongly claimed, as only four ARGs were taken into count and no serotyping was performed. However, the presence of multiple ARGs in STEC isolates indicated the presence of multidrug resistance in the identified STEC isolates.

The antimicrobial susceptibility test of STEC isolates in our study showed that all the STEC isolates (*n* = 16 out of 16) were MDR (Figure 5). We found no studies on the AMR profile of STEC isolated from SCM milk in Bangladesh. A few studies have reported the presence of MDR‐ *E. coli* in raw milk in Bangladesh (Haque et al. 2014; Rahman et al. 2017), and one of these studies reported about 28.13% prevalence (Rahman et al. 2017). However, outside Bangladesh, a study reported 79.48% MDR‐STEC prevalence in bovine SCM milk (Ahmadi et al. 2020). Another study reported 100% multidrug resistance in STEC isolated from bovine mastitis, which supports the current findings (Tavakoli and Pourtaghi, 2017). Hence, we conclude that STEC isolated from SCM milk is predominantly multidrug resistant.

The current study findings also depict that MDR‐STEC isolates were fully resistant to amoxicillin and highly resistant to common antibiotics such as ampicillin, gentamycin, and tetracycline (Figure 5). A previous study conducted in Bangladesh reported complete resistance of *E. coli* (did not identify STEC) isolated from SCM milk against amoxicillin and ampicillin (Haque et al. 2014). Other studies conducted on *E. coli* isolated from bovine mastitis in Bangladesh, also reported high resistance against tetracycline (89.5%), ampicillin (89.5%), and amoxicillin (94.5%) (Bag et al. 2021). In Iran, a study on STEC from SCM milk reported high resistance to tetracycline and ampicillin, which aligns with the findings of the current study. However, 50% of the STEC were susceptible to gentamicin, though the susceptibility varied depending on the STEC serotypes (Ahmadi et al. 2020). Additionally, a few studies in Bangladesh also reported *E. coli* susceptibility to gentamycin and tetracycline (Farhad et al. 2021; Islam et al. 2016). Contrary, a study found that 98.03% of STEC isolates from human, animal, and food sources had resistance against gentamycin (Rubab and Oh, 2020). Based on the current study and previous study findings, it is evident that the commonly used antibiotics have already been resistant, though few previous studies found gentamycin to be susceptible (Ahmadi et al. 2020; Farhad et al. 2021; Islam et al. 2016). In this way, resistant pathogens can enter the food chain not only via milk but also through other animal‐origin foods, for example, the presence of resistant *E. coli* in beef and poultry has also been reported in Bangladesh (Hossain et al. 2008; Rana, Chowdhury, et al. 2024). Moreover, in our previous studies, we have reported the zoonotic linkage of resistant pathogens in dairy farms (Roy et al. 2024), and also found that biosecurity practices in dairy farms can be potentially associated with human health (Chowdhury et al. 2023). Hence, the possibility of MDR‐STEC entering human food chain via animal origin food is obvious if proper safety and precautions are not taken in the first place.

Moreover, the current study result depicts that the *stx1* gene of isolated STEC from SCM milk is closely related to different strains of STEC from cattle, humans, food, human stool, and cattle feces origins (Figure 6). These strains were mostly non‐O157, except for two O157 strains of cattle and cattle feces origins. Previous studies also reported non‐O157 serotypes to be predominant in cattle (Pradel et al. 2000; Torres et al. 2018). Additionally, our study sequence showed close relativeness with a strain BAU‐MH1 (KM596779.1), which was isolated from rectal swabs of apparently healthy cattle in Bangladesh (Hassan et al. 2017). We also found similarities with human‐origin strains such as O152_EC108 (EU754740.1) and OUT_EC120 (EU754738.1) that were associated with diseases (Rui et al. 2010). Moreover, several STEC serotypes found in lactating dairy cattle were reported to be associated with human diseases (Ballem et al. 2020). Additionally, strains such as O88:H38_N2688 (GQ429156.1) and O8:H16_20177 (GQ429154.1), isolated from beef meat, were also found to be closely related to our study sequence (Xia et al. 2010). The presence of MDR‐STEC in milk, with similarities to disease‐causing strains, should be considered a potential threat for foodborne pathogen outbreaks if proper precautions are not taken. However, we cannot confirm the zoonotic pathway of MDR‐STEC with our current findings. A thorough investigation is needed on the whole dairy supply chain to ensure the pathway of MDR‐STEC spreading from milk.

## 5 Conclusions

We found SCM to be highly prevalent in dairy cattle, with a significant presence of STEC. The STEC isolates contained *stx1, stx2*, and *eaeA* virulence genes. All STEC isolates showed multidrug resistance and harbored ARGs as well. Additionally, the isolated MDR‐STEC carried a gene that produces the Shiga toxin‐1 that was similar to several disease‐causing strains linked to cattle, food, and humans. This suggests that SCM milk may serve as a vehicle for the spread of zoonotic MDR‐STEC infections. We need to consider this when implementing appropriate control and preventative measures and creating long‐term strategies to ensure the safety of bovine milk‐derived food.

## Author Contributions

**Tonmoy Chowdhury:** conceptualization (equal), data curation (equal), formal analysis (equal), investigation (equal), methodology (equal), software (equal), writing – original draft (equal). **Mithu Chandra Roy:** conceptualization (equal), investigation (equal), methodology (equal), software (equal). **Ferdaus Mohd Altaf Hossain:** conceptualization (equal), project administration (equal), supervision (equal), writing – review and editing (equal).

## Ethics Statement

The study was conducted under the supervision of the Department of Dairy Science, Faculty of Veterinary, Animal and Biomedical Sciences, Sylhet Agricultural University, Sylhet‐3100, Bangladesh. International standards considering animal welfare, laboratory hygiene, and ethics were maintained from the start to the end of this study. Approval for conducting the current study was taken from the respective concerned authority of the university.

## Conflicts of Interest

The authors declare no conflicts of interest.

## Supporting information


Data S1.

## Data Availability

The data that support the findings of this study are available on request from the corresponding author.
