# Supplementary material for: Prevalence and Zoonotic Risk of Multidrug‐Resistant Escherichia coli in Bovine Subclinical Mastitis Milk: Insights Into the Virulence and Antimicrobial Resistance
Source: Food Sci Nutr. 2025 Jan 15;13(1):e4761. doi: 10.1002/fsn3.4761 (PMC11733599; doi:10.1002/fsn3.4761)
Supplement: Supplementary file 1 — Data S1. [file FSN3-13-e4761-s001.docx]

**SUPPLEMENTARY TABLES**

**Table 1: List of antibiotics used for antimicrobial susceptibility test.**

| Antibiotic Groups | Names of Antibiotics |
| --- | --- |
| Penicillin | Ampicillin (AMP, 10μg), Amoxicillin (AMX, 30μg), Oxacillin (OX, 1μg) |
| Cephalosporins | Ceftazidime (CAZ, 30μg), Cefotaxime (CTX, 30μg), Cefoxitin (CX, 30μg) |
| Tetracyclines | Tetracycline (TE, 30μg) |
| Aminoglycosides | Gentamicin (GEN, 10μg), Streptomycin (S, 10μg) |
| Carbapenem | Meropenem (MEM, 10μg) |
| Monobactam | Aztreonam (AT, 30μg) |
| Glycopeptide | Vancomycin (VA, 30μg) |
| Aminocoumarin | Novobiocin (NV, 30µg) |

**Table 2: Different characteristics of presumptively confirmed *E. coli* isolates found in cultural media and biochemical tests in the current study.**

| **Cultural and Biochemical tests** | | **Characteristics of *E. coli* isolates** |
| --- | --- | --- |
| EMB | | Colonies with dark center and green metallic sheen |
| Gram Staining | | Gram (-ve), rod-shaped, pink color organism |
| Motility Indole Urea test | Motility test | +ve (uniform turbidity in medium) |
|  | Indole test | +ve (cherry-red color ring) |
|  | Urea test | -ve |
| Methyl Red test | | +ve (pink-red color development) |
| Voges-Proskauer test | | -ve (no color change) |
| Citrate test | | -ve (no change of color) |
| Growth on Sugar fermentation test (TSI) | Butt | Yellow/Acidic |
|  | Slant | Yellow/Acidic |
|  | Gas | +ve (presence of gas bubble) |
|  | H_2_S | -ve |
| Catalase test | | +ve |
